# Explainable machine learning for the early differentiation of pediatric bronchopneumonia using routine laboratory parameters

**DOI:** 10.1371/journal.pone.0351509

**Published:** 2026-07-08

**Authors:** Jinxing Dai, Hao Qiu, Liran Shen, Qianjin Shi, Kang Shen, Qingkai Wang, Weibing Qiu

**Affiliations:** 1 Department of Pediatrics, Siyang Hospital, Suqian, China; 2 Department of Radiation Oncology, Siyang Hospital, Suqian, China; 3 Department of Clinical Laboratory, Shanxian Central Hospital, Heze, China; Children’s National Hospital, George Washington University, UNITED STATES OF AMERICA

## Abstract

**Objective:**

Early differentiation of pediatric acute respiratory infections in outpatient and emergency settings is often hindered by nonspecific clinical signs, leading to unnecessary empirical antibiotic use and avoidable radiation exposure. Therefore, this study aimed to develop and internally evaluate an early triage model to distinguish pediatric bronchopneumonia (BP) from uncomplicated upper respiratory tract infection using routine, cost-effective laboratory parameters analyzed with machine-learning algorithms.

**Methods:**

This retrospective study consecutively enrolled 532 pediatric patients who presented with mild respiratory symptoms at their initial visit, comprising 218 in the BP group and 314 in the Upper Respiratory Tract Infection (URTI) group. Core laboratory indicators were selected using a dual-dimensionality reduction strategy that integrated the Least Absolute Shrinkage and Selection Operator (LASSO) regression with the Boruta algorithm. Seven machine learning classifiers were then constructed and compared based on the resulting feature matrix. The Shapley Additive Explanations (SHAP) framework was subsequently applied to interpret the nonlinear predictive mechanisms of the optimal model.

**Results:**

This study constructed seven machine learning models: Logistic Regression (LR), Decision Tree (DT), Random Forest (RF), Extreme Gradient Boosting (XGBoost), Lightweight Gradient Boosting Machine (LightGBM), Support Vector Machine (SVM), and Artificial Neural Network (ANN). In the internal validation set, the SVM model demonstrated favorable predictive performance, with an area under the curve (AUC) of 0.921 (95% confidence interval: 0.874–0.959). Based on Platt-scaled probability estimates, the SVM model showed the lowest Brier score among the evaluated models, with a Brier score of 0.112. Decision curve analysis confirmed this model’s positive net clinical benefit across a broad range of threshold probabilities. SHAP analysis further elucidated the nonlinear contribution weights of multiple conventional parameters at specific physiological thresholds.

**Conclusions:**

The multidimensional SVM risk quantification model, based on nine routine laboratory parameters, provides an accurate and objective assessment of pediatric BP risk. This model holds significant potential for clinical translation as a noninvasive, cost-effective triage tool in emergency departments. Its application could effectively reduce unnecessary radiographic screening and excessive antibiotic use.

## Introduction

Bronchopneumonia (BP) is one of the leading causes of pediatric hospitalizations worldwide [1,2]. The disease typically presents with an insidious onset. It lacks specific early clinical features, frequently leading to diagnostic confusion with self-limiting uncomplicated upper respiratory tract infection (URTI) at initial presentation [3,4]. To prevent occult disease progression, broad-spectrum antibiotics are frequently prescribed prophylactically or empirically in routine clinical practice [5]. However, amid the escalating global crisis of antimicrobial resistance (AMR), a critical bottleneck in pediatric practice remains: accurately identifying genuinely low-risk patients who do not require antibiotic exposure or radiographic imaging during initial emergency department observation or hospital admission, which is essential to facilitate safe therapeutic de-escalation.

Currently, the definitive diagnosis of BP relies on chest imaging and etiological evaluation [6]. Pediatric BP has a heterogeneous etiological spectrum. Respiratory viruses and bacterial pathogens are the most common causes, whereas atypical organisms and hospital-acquired Gram-negative bacteria may also contribute, particularly in older children, immunocompromised patients, and healthcare-associated cases. Although culture-based bacterial identification and antimicrobial susceptibility testing (AST) remain essential for etiological confirmation and targeted antimicrobial therapy, these methods are time-consuming. Moreover, because pediatric respiratory samples are often complex and may contain mixed flora or low pathogen loads, rapid and reliable bacterial identification and AST remain challenging [7]. Molecular diagnostic methods, including isothermal nucleic acid amplification and CRISPR-Cas-based detection strategies, have become increasingly attractive for infectious disease diagnostics; however, their turnaround time, technical requirements, and accessibility may still limit their immediate use in early outpatient or emergency triage settings [8]. However, in high-volume primary or emergency care settings, radiographic imaging is often deliberately deferred to strictly limit pediatric exposure to ionizing radiation. Concurrently, the prolonged turnaround time for etiological assays forces physicians to make empirical treatment decisions in the initial hours of observation. Although routine inflammatory biomarkers serve as the cornerstone of clinical assessment and possess irreplaceable value in identifying severe bacterial infections [9,10], they exhibit extensive overlapping in normal reference intervals when differentiating early, mild respiratory symptoms—a recognized “clinical gray zone.” Consequently, the isolated use of single indicators fails to afford clinicians sufficient diagnostic confidence to safely rule out the risk of pneumonia [11,12]. This inherent diagnostic uncertainty directly drives the persistent overprescription of empirical antibiotics.

Comprehensive routine peripheral blood panels, characterized by their minimally invasive, reproducible, and highly standardized nature, enable deep characterization of the host’s systemic stress response during the early phase of infection [13,14]. Beyond traditional fluctuations in inflammatory cell populations, the infection-induced acute-phase response triggers profound metabolic restructuring and reprogramming of nutritional immunity. For instance, acute lower respiratory tract infections are not only accompanied by the rapid mobilization of humoral immunity (e.g., IgA, IgG, IgM) but also frequently induce the redistribution of serum trace elements (e.g., Cu, Zn, Fe) and metabolic stress, as evidenced by alterations in liver enzymes and lactate dehydrogenase [15–17].

Nonetheless, pediatric clinical biochemical and immunological parameters are highly age-dependent and exhibit complex nonlinear interactions throughout disease evolution [18]. Traditional linear statistical models are intrinsically inadequate for extracting robust features to enable precise “rule-out” diagnostics in such a high-dimensional, dynamic physiological landscape [19]. Recently, machine learning (ML)—leveraging its superior capacity for feature space representation and nonlinear fitting—has emerged as an ideal methodological framework for constructing clinical decision support systems with high negative predictive values (NPVs) [20]. Specifically, tree-based ensemble algorithms can adeptly capture the intricate coupling between subtle multidimensional biochemical perturbations and patient baseline characteristics, thereby delineating highly precise risk boundaries [21].

Against this backdrop, the present study leveraged a merged dual-center retrospective cohort from Siyang Hospital and Shanxian Central Hospital to develop and internally evaluate a series of machine learning models. By integrating multidimensional routine peripheral blood profiles, we aimed to estimate the risk of BP in pediatric patients presenting with mild respiratory symptoms. Model performance was assessed using an internal validation set randomly derived from the merged dual-center cohort.

## Materials and methods

### Study design and participants

This retrospective case-control study consecutively enrolled pediatric patients who presented to the pediatric departments of Siyang Hospital and Shanxian Central Hospital between April 15, 2021, and January 20, 2025. All research procedures involving human participants adhered to the 1964 Declaration of Helsinki and its subsequent revisions or equivalent ethical standards. This study was approved by the Medical Ethics Committee of Siyang Hospital, approval number KS2026002. Given the retrospective nature of the study and the use of de-identified data, the requirement for informed consent was waived. De-identified data were accessed for research purposes on January 5, 2026. As only de-identified data were used for statistical analysis, the authors had no access to information that could directly identify individual participants at any stage of the study.

According to the final outpatient or emergency department diagnoses, the study cohort was stratified into a BP group and an uncomplicated URTI control group. The URTI group was strictly defined as patients presenting with mild respiratory symptoms at initial evaluation who were ultimately diagnosed with a common URTI and remained free of antibiotic exposure throughout their entire clinical course.

The inclusion criteria were as follows: (1) age ranging from 28 days to 14 years; (2) for the BP group, fulfillment of current clinical and radiographic diagnostic criteria for pediatric BP at their initial presentation; and (3) peripheral blood sampling performed at the initial emergency visit, strictly before the administration of any empirical antibiotics or specific therapies.

Patients were excluded if they met any of the following criteria: (1) underlying chronic cardiopulmonary conditions (e.g., congenital heart disease, bronchopulmonary dysplasia [BPD]) or inherited metabolic disorders; (2) primary or secondary immunodeficiency, including a history of autoimmune diseases or long-term immunosuppressant therapy; or (3) concomitant hematologic malignancies or severe organ dysfunction.

### Clinical feature space and multiple imputation

Demographic characteristics and laboratory test results at initial presentation were retrospectively extracted from the dual-center electronic medical record (EMR) systems. The extracted feature variables encompassed baseline demographics, routine blood indices, biochemical markers, serum trace elements, and immunoglobulins.

To address missing data for specific laboratory parameters, we used Multiple Imputation by Chained Equations (MICE) under the missing-at-random (MAR) assumption within the training set. Specifically, missing continuous variables in the training set were imputed using the predictive mean matching (PMM) algorithm. The imputation procedure fitted in the training set was subsequently applied to the internal validation set. A total of five imputed training datasets were generated and subsequently pooled to facilitate downstream feature selection and model construction.

### Feature selection

To mitigate multicollinearity in high-dimensional laboratory data and prevent model overfitting, we employed a dual feature selection strategy that integrates Least Absolute Shrinkage and Selection Operator (LASSO) regression and the Boruta algorithm. Initially, LASSO regression was applied to the imputed training set for preliminary dimensionality reduction. The optimal penalty parameter (λ) was determined via 10-fold cross-validation to shrink coefficients and eliminate redundant linearly correlated variables. Concurrently, to overcome the intrinsic limitations of linear algorithms in capturing nonlinear interactions, we implemented the Boruta algorithm—rooted in a random forest architecture—to evaluate and extract globally important features via permutation tests. To ensure the stability and robustness of the feature selection, we derived the intersection of variables retaining non-zero coefficients in LASSO and those classified as “Confirmed” by Boruta. This intersection was ultimately established as the core feature subset, serving as the exclusive input matrix for all downstream machine learning predictive models.

### Machine learning model development and training

The merged dual-center dataset (N = 532) was randomly split at a 7:3 ratio into a training set (n = 373) and an internal validation set (n = 159). All preprocessing steps, including MICE imputation, feature selection, hyperparameter tuning, and model training, were performed within the training set. The internal validation set was used only for final model evaluation. Using the feature subset derived from the aforementioned feature selection strategy, we trained seven machine learning classification algorithms on the training set: Logistic Regression (LR), Decision Tree (DT), Random Forest (RF), Extreme Gradient Boosting (XGBoost), Lightweight Gradient Boosting Machine (LightGBM), Support Vector Machine (SVM), and Artificial Neural Network (ANN). Table 1 presents the optimal hyperparameter combinations for each model.

**Table 1:** Optimal parameter combination for machine learning models.

Model	Optimal hyperparameters
Decision Tree	ccp_alpha = 0.0; max_depth = None; max_features = None; min_samples_split = 10
Random Forest	n_estimators = 350; max_features = 2
XGBoost	learning_rate = 0.2; max_depth = 5; n_estimators = 100; subsample = 0.8
LightGBM	colsample_bytree = 1.0; learning_rate = 0.2; n_estimators = 100; num_leaves = 31; subsample = 0.6
SVM	svc__C = 100; svc__gamma = 0.01; svc__kernel = ‘rbf’
ANN	activation = ‘tanh’; hidden_layer_sizes = 50

XGBoost, Extreme Gradient Boosting, LightGBM, Light Gradient Boosting Machine, SVM Support Vector Machine, ANN Artificial Neural Network.

Grid search was selected for hyperparameter optimization because the number of candidate predictors after feature selection was limited and the predefined hyperparameter space for each algorithm was relatively small, making this approach transparent, reproducible, and computationally feasible. During model training, 10-fold cross-validation was used for all algorithms to mitigate overfitting and ensure internal stability. Concurrently, grid search was used to iteratively tune hyperparameters and determine the optimal configuration for each algorithm. The SVM model used a radial basis function (RBF) kernel, with optimized hyperparameters of C = 100 and gamma = 0.01, allowing the model to capture nonlinear decision boundaries and non-monotonic relationships among laboratory parameters. For the SVM model, probability estimates were obtained using Platt scaling based on the training data. Subsequently, the seven hyperparameter-optimized models were evaluated in the internal validation set to assess their internal predictive performance.

### Model evaluation and statistical analysis

Continuous variables conforming to a normal distribution were expressed as the mean ± standard deviation (SD) and compared using the independent-samples t-test. In contrast, non-normally distributed variables were presented as the median (interquartile range [IQR]) and analyzed via the Wilcoxon rank-sum test. Categorical variables were summarized as frequencies (percentages) and compared using either Pearson’s chi-square test or Fisher’s exact test, as appropriate.

To evaluate the internal predictive performance of the models in the internal validation set, we assessed them across three distinct dimensions: discrimination, calibration, and clinical utility. Discrimination was comprehensively assessed utilizing the area under the receiver operating characteristic curve (AUC), accuracy, sensitivity, specificity, positive predictive value (PPV), negative predictive value (NPV), F1 score, and Cohen’s kappa coefficient. Calibration was quantified using Brier scores and visualized by plotting calibration curves. For the SVM model, Brier scores, calibration curves, and decision curve analysis were based on the Platt-scaled probability estimates. For clinical utility, we applied decision curve analysis (DCA) to estimate the net clinical benefit across a continuum of threshold probabilities, and we mapped the distribution of classification errors using confusion matrices. Furthermore, the Shapley Additive Explanations (SHAP) framework was used to elucidate the model’s interpretability. In the SHAP analysis, bronchopneumonia was defined as the positive class (Class 1), whereas uncomplicated URTI was defined as Class 0.

All fundamental statistical analyses were performed using SPSS version 27.0 and R version 4.5.1. Multiple imputation was performed using the mice package in R. Feature selection was conducted using the glmnet and Boruta packages in R. Machine learning model development, hyperparameter tuning, probability calibration, and model evaluation were performed in Python 3.12 using scikit-learn, XGBoost, and LightGBM. SHAP interpretation was performed using the SHAP package in Python. A two-sided P-value < 0.05 was defined as indicating statistical significance.

## Results and discussion

### Patient characteristics

A total of 532 pediatric patients were enrolled in this study, including 366 males and 166 females. Based on their final clinical diagnoses, the cohort was stratified into a BP group (n = 218) and an uncomplicated URTI control group (n = 314). Age was further categorized into three groups: < 5 years, ≥ 5 and ≤10 years, and >10 years. Overall, 69 patients (12.97%) were younger than 5 years, 278 patients (52.26%) were aged ≥5 and ≤10 years, and 185 patients (34.77%) were older than 10 years. The distribution of age categories differed significantly between the BP and URTI groups (P < 0.001), whereas sex distribution showed no significant difference between the two groups (P = 0.637) (Table 2).

**Table 2:** Baseline characteristics.

Variables	Total(N = 532)	uncomplicated URTI (N = 314)	Bronchopneumonia (N = 218)	P-Value
Sex (%)				0.637
Female	166 (31.20)	95 (30.25)	71 (32.57)	
Male	366 (68.80)	219 (69.75)	147 (67.43)	
Age(years)				<0.001
<5	69 (12.97)	26 (8.28)	43 (19.72)	
≥5, ≤ 10	278 (52.26)	187 (59.55)	91 (41.74)	
>10	185 (34.77)	101 (32.17)	84 (38.53)	
WBC (10^9/L)	8.34 ± 1.52	8.17 ± 1.20	8.58 ± 1.86	0.002
NEUT (10^9/L)	3.13 [2.63, 3.64]	3.06 [2.71, 3.51]	3.23 [2.48, 4.06]	0.028
LYMPH (10^9/L)	3.87 ± 0.96	3.99 ± 0.95	3.71 ± 0.95	0.001
MONO (10^9/L)	0.64 ± 0.21	0.65 ± 0.19	0.61 ± 0.23	0.025
RBC (10^12/L)	4.38 [4.20, 4.53]	4.43 [4.29, 4.60]	4.25 [4.10, 4.44]	<0.001
PLT (10^9/L)	307.50 [271.00, 342.25]	317.00 [279.00, 346.75]	293.00 [259.25, 335.00]	<0.001
CRP (mg/L)	4.60 [3.66, 5.79]	4.52 [3.88, 5.32]	4.96 [3.13, 7.08]	0.036
ALT (U/L)	16.00 [14.00, 18.00]	16.00 [14.00, 18.00]	16.00 [14.00, 18.00]	0.939
ALP (U/L)	189.00 [165.00, 216.00]	183.00 [163.00, 207.00]	198.50 [169.00, 220.75]	<0.001
GGT (U/L)	13.00 [11.00, 17.00]	12.00 [10.00, 13.00]	17.00 [15.00, 19.00]	<0.001
ALB (g/L)	41.80 [40.30, 42.92]	42.60 [41.62, 43.50]	40.30 [39.12, 41.30]	<0.001
TBA (μmol/L)	4.40 [2.77, 6.00]	5.30 [3.90, 6.68]	2.90 [2.40, 4.65]	<0.001
ADA (U/L)	14.10 [12.30, 15.90]	13.75 [12.00, 15.20]	14.80 [12.90, 16.80]	<0.001
UREA (mmol/L)	2.50 [2.10, 3.30]	2.10 [1.90, 2.40]	3.25 [2.90, 3.50]	<0.001
CREA (μmol/L)	28.10 ± 5.66	27.11 ± 5.66	29.51 ± 5.36	<0.001
UA (μmol/L)	210.00 [181.75, 237.00]	205.50 [181.00, 232.75]	217.00 [183.50, 244.00]	0.018
LDH (U/L)	236.68 ± 17.01	232.29 ± 15.18	243.01 ± 17.52	<0.001
CK (U/L)	97.05 ± 30.18	94.28 ± 30.99	101.03 ± 28.59	0.011
GLDH (U/L)	1.80 [1.50, 2.30]	1.60 [1.30, 1.90]	2.30 [1.90, 2.70]	<0.001
HCY (μmol/L)	5.00 [4.00, 6.00]	5.00 [4.00, 6.00]	5.00 [4.25, 6.75]	0.617
NEFA (mmol/L)	0.33 [0.23, 0.47]	0.29 [0.21, 0.39]	0.42 [0.30, 0.55]	<0.001
SA (mg/dL)	54.69 ± 6.63	54.51 ± 6.82	54.96 ± 6.35	0.443
CG (mg/dL)	0.90 [0.60, 1.20]	0.80 [0.60, 1.00]	1.10 [0.80, 1.40]	<0.001
RBP (mg/dL)	18.30 ± 2.41	18.33 ± 2.35	18.25 ± 2.49	0.689
Cu (μmol/L)	21.58 ± 5.48	21.65 ± 5.52	21.49 ± 5.45	0.748
Zn (μmol/L)	11.60 [10.30, 12.90]	12.40 [11.43, 13.50]	10.15 [8.90, 11.28]	<0.001
Fe (μmol/L)	12.34 ± 1.65	12.55 ± 1.54	12.04 ± 1.76	<0.001
IMA (U/mL)	71.70 [70.47, 74.70]	70.60 [69.90, 71.38]	75.00 [74.32, 75.60]	<0.001
IgA (g/L)	0.97 [0.79, 1.19]	0.86 [0.73, 1.00]	1.21 [0.98, 1.46]	<0.001
IgG (g/L)	7.37 ± 2.17	7.69 ± 2.15	6.90 ± 2.13	<0.001
IgM (g/L)	1.12 [0.85, 1.34]	1.05 [0.80, 1.28]	1.19 [0.93, 1.50]	<0.001

WBC, white blood cell count; NEUT, neutrophil count; LYMPH, lymphocyte count; MONO, monocyte count; RBC, red blood cell count; PLT, platelet count; CRP, C-reactive protein; ALT, alanine aminotransferase; ALP, alkaline phosphatase; GGT, gamma-glutamyl transferase; ALB, albumin; TBA, total bile acid; ADA, adenosine deaminase; UREA, urea; CREA, creatinine; UA, uric acid; LDH, lactate dehydrogenase; CK, creatine kinase; GLDH, glutamate dehydrogenase; HCY, homocysteine; NEFA, non-esterified fatty acids; SA, sialic acid; CG, cholylglycine; RBP, retinol-binding protein; Cu, copper; Zn, zinc; Fe, iron; IMA, ischemia-modified albumin; IgA, immunoglobulin A; IgG, immunoglobulin G; IgM, immunoglobulin M.

Compared to the URTI group, patients in the BP group exhibited significantly elevated baseline levels of white blood cell count (WBC), neutrophil count (NEUT), C-reactive protein (CRP), alkaline phosphatase (ALP), gamma-glutamyl transferase (GGT), adenosine deaminase (ADA), urea (UREA), creatinine (CREA), uric acid (UA), lactate dehydrogenase (LDH), creatine kinase (CK), glutamate dehydrogenase (GLDH), non-esterified fatty acids (NEFA), ischemia-modified albumin (IMA), cholylglycine (CG), immunoglobulin A (IgA), and immunoglobulin M (IgM) (all P < 0.05). Conversely, the BP group demonstrated significantly decreased levels of lymphocyte count (LYMPH), monocyte count (MONO), red blood cell count (RBC), platelet count (PLT), albumin (ALB), total bile acids (TBA), zinc (Zn), iron (Fe), and immunoglobulin G (IgG) (all P < 0.05). No significant between-group differences were observed regarding homocysteine (HCY), sialic acid (SA), retinol-binding protein (RBP), alanine aminotransferase (ALT), and copper (Cu) (all P > 0.05) (Table 2).

### Select feature variables for model construction

**Fig 1:**
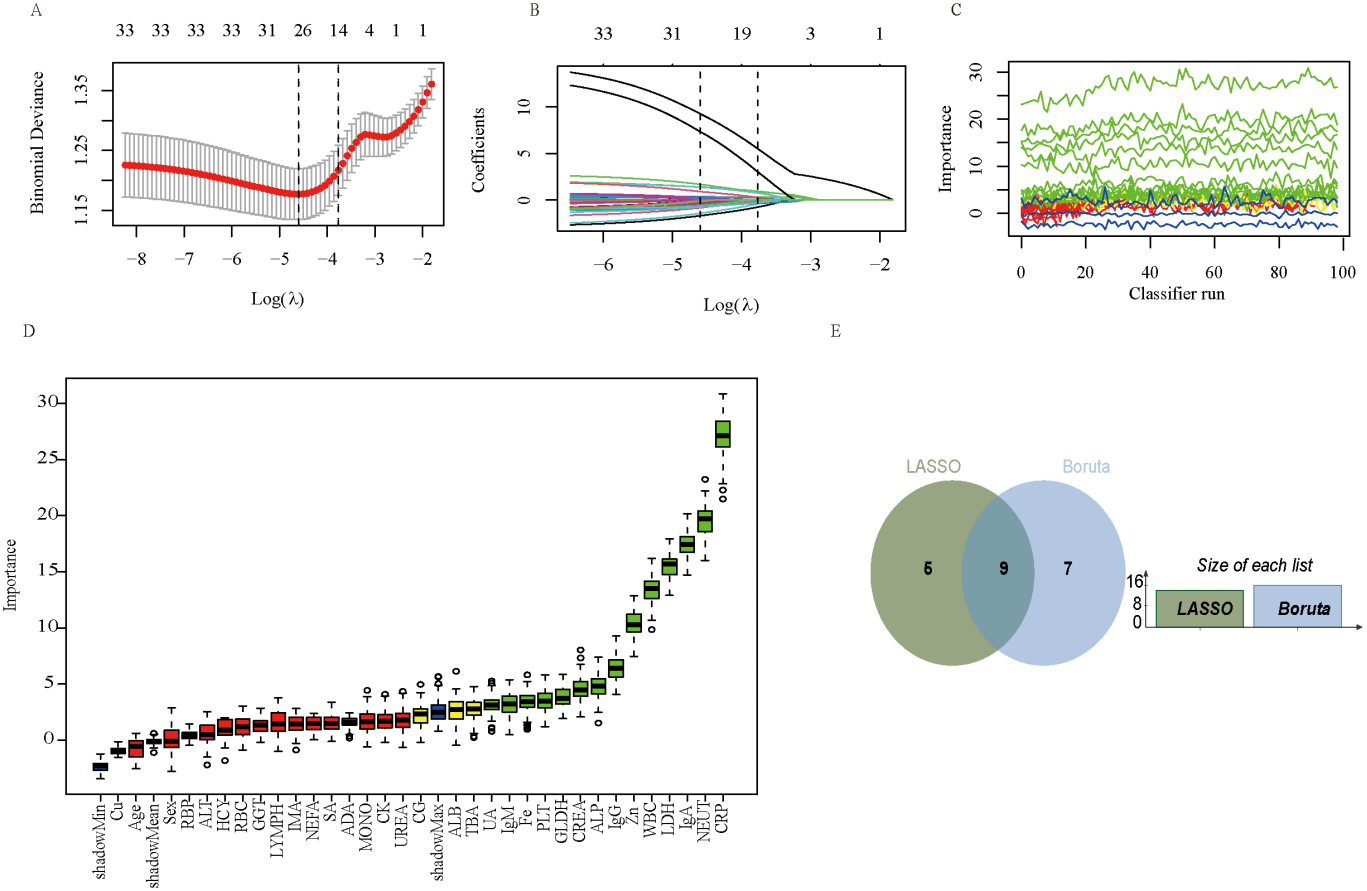
Feature selection. (A) LASSO cross-validation curve. The two dashed vertical lines indicate λmin, corresponding to the minimum cross-validation error, and λ1se, corresponding to the largest λ within one standard error of the minimum. The red dots represent the mean cross-validation error, the gray error bars indicate ±1 standard error, and the numbers above the curve denote the number of non-zero coefficients. (B) LASSO coefficient path showing the shrinkage trajectories of regression coefficients for different variables as a function of log(λ). (C) Changes in variable importance across repeated Boruta training iterations, used to evaluate the stability of variable importance and distinguish important variables from shadow features. (D) Box plot of the variable importance distribution generated by the Boruta algorithm. (E) Network diagram showing the overlapping and selected features identified by LASSO and Boruta.

In this study, we employed LASSO regression and the Boruta algorithm for rigorous feature selection. During the LASSO analysis, the optimal penalty parameter (λ) was determined via cross-validation (Fig 1A). As λ increased, model complexity progressively decreased. By applying the 1-standard error (1-SE) criterion to minimize the binomial deviance, 14 feature variables were retained from the 33 initial candidates. The coefficient profile plot against log(λ) revealed that specific variables maintained stable predictive contributions even under stringent regularization conditions (Fig 1B).

Subsequently, we applied the random forest-based Boruta algorithm to evaluate variable robustness from the perspective of predictive importance. The importance scores of authentic variables were consistently higher than those of the shadow (permuted) control features (Fig 1C), indicating that the selection outcomes were driven by genuine signals rather than statistical noise. The distribution of variable importance exhibited distinct gradients (Fig 1D); variables including CRP, NEUT, IgA, LDH, and WBC occupied the upper echelon of importance, whereas the shadow control features (shadow Min, shadow Mean, and shadow Max) ranked the lowest, aligning perfectly with algorithmic expectations. The feature selection outcomes of both methodologies demonstrated robust consistency: LASSO identified 14 features, Boruta confirmed 16 features, and their intersection yielded a consensus of 9 core features (Fig 1E). Synthesizing the linear interpretability of LASSO and the robustness of Boruta to identify nonlinear contributions, we exclusively used the intersected feature subset for all downstream model construction and analyses.

### Model evaluation

**Fig 2:**
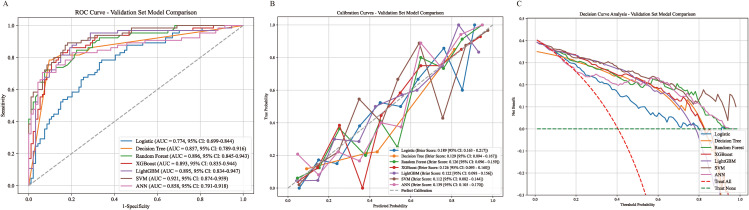
Model performance evaluation. (A) ROC Curves for the Models. (B) Calibration curves for the models. (C) Decision curve analysis curves for the models.

Using the training cohort, we developed seven machine learning models—LR, DT, RF, XGBoost, LightGBM, SVM, and ANN—and subsequently evaluated their predictive performance in the internal validation set. Receiver operating characteristic (ROC) analysis revealed that the SVM model achieved the highest area under the curve (AUC) of 0.921 (95% CI: 0.874–0.959) in the internal validation set, followed by RF (AUC = 0.896, 95% CI: 0.845–0.943), LightGBM (AUC = 0.895, 95% CI: 0.834–0.947), and XGBoost (AUC = 0.893, 95% CI: 0.835–0.944). Conversely, LR exhibited a comparatively lower AUC of 0.774 (95% CI: 0.699–0.844) (Fig 2A).

**Fig 3:**
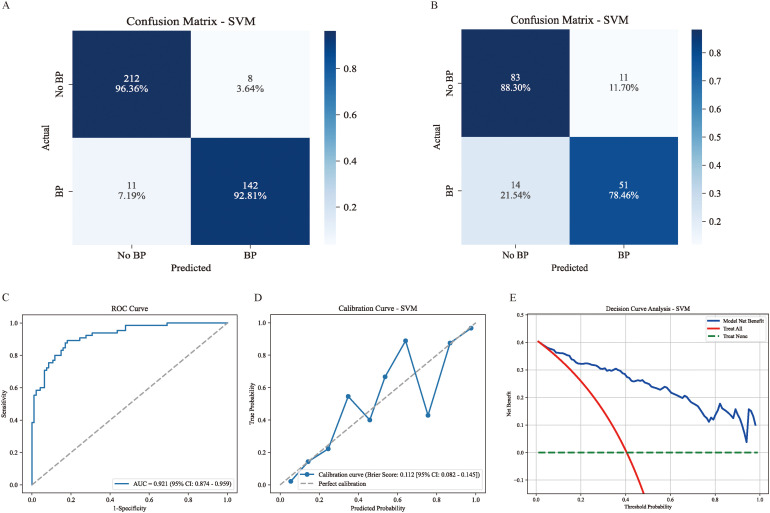
Performance evaluation of the SVM model in the internal validation set. (A) Training set confusion matrix. (B) Internal validation set confusion matrix. (C) ROC curve of the SVM model in the internal validation set. (D) Calibration curve of the SVM model in the internal validation set. (E) Decision curve analysis of the SVM model in the internal validation set.

Upon comprehensive evaluation of further performance metrics in the internal validation set, the SVM model consistently outperformed the others, yielding an accuracy of 0.843, precision of 0.823, sensitivity of 0.785, specificity of 0.883, F1 score of 0.803, positive predictive value (PPV) of 0.823, and negative predictive value (NPV) of 0.856, thereby demonstrating optimal classification efficacy (Table 3). Based on Platt-scaled probability estimates, calibration curves and Brier scores showed that the SVM model had the lowest Brier score in the internal validation set (0.112, 95% CI: 0.082–0.144), indicating the lowest overall probabilistic prediction error among the evaluated models. LightGBM also demonstrated a relatively low Brier score (0.120, 95% CI: 0.093–0.156), whereas LR yielded a notably higher Brier score (0.189, 95% CI: 0.163–0.217) (Fig 2B). DCA indicated that, across a broad range of threshold probabilities, most models achieved a higher net clinical benefit than both the “treat-all” and “treat-none” strategies. Notably, the DCA curve for SVM was positioned at a higher overall level, suggesting its superior potential value for clinical decision-making within the corresponding threshold intervals (Fig 2C). Confusion matrix analysis revealed that the SVM model’s false positive and false negative rates on the training set were 3.64% and 7.19%, respectively (Fig 3A). In the internal validation set, these rates were 11.70% and 21.54%, respectively (Fig 3B), indicating its classification performance in the internal validation cohort. Furthermore, the detailed presentation of the ROC, calibration, and DCA curves for the SVM model in the internal validation set further illustrated its discrimination, calibration, and potential clinical utility in this internal evaluation (Fig 3C–E).

**Table 3:** Comparative analysis of the performance outcomes across machine learning models.

Model	AUC	Accuracy	Precision	Sensitivity	Specificity	F1 Score	Kappa	Youden’s J	PPV	NPV
Logistic	0.774	0.698	0.613	0.708	0.691	0.657	0.390	0.399	0.613	0.774
Decision Tree	0.857	0.799	0.726	0.815	0.787	0.768	0.591	0.603	0.726	0.860
Random Forest	0.896	0.805	0.724	0.846	0.777	0.780	0.607	0.623	0.724	0.880
XGBoost	0.893	0.824	0.747	0.862	0.798	0.800	0.644	0.659	0.747	0.893
LightGBM	0.895	0.818	0.750	0.831	0.809	0.788	0.629	0.639	0.750	0.874
SVM	0.921	0.843	0.823	0.785	0.883	0.803	0.672	0.668	0.823	0.856
ANN	0.858	0.805	0.750	0.785	0.819	0.767	0.599	0.604	0.750	0.846

XGBoost, Extreme Gradient Boosting, LightGBM, Light Gradient Boosting Machine, SVM, Support Vector Machine, ANN, Artificial Neural Network, PPV, Predictive Value, NPV, Negative Predictive Value, AUC, Area Under the Curve.

### Interpretability analysis in the model

**Fig 4:**
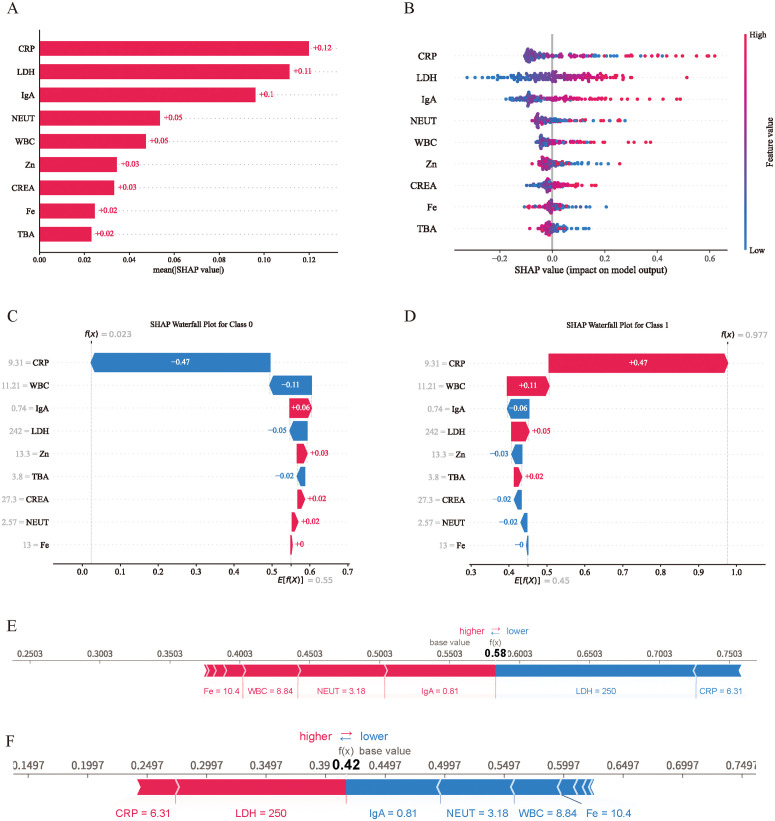
SHAP explains the results of the SVM model (A) Global feature importance ranked by mean(|SHAP|), summarizing the average magnitude of each feature’s contribution to the model output. (B) SHAP beeswarm plot: SHAP values (x-axis) quantify each feature’s impact on the model output, and color encodes feature values from low to high, illustrating how value ranges relate to prediction direction. (C–D) SHAP waterfall plots for the same sample showing the feature contributions for Class 1 (bronchopneumonia) and Class 0 (uncomplicated URTI), respectively. (E–F) SHAP force plots for the same sample showing the feature contributions for Class 1 (bronchopneumonia) and Class 0 (uncomplicated URTI), respectively.

To elucidate the predictive rationale of the optimal SVM model for BP incidence, we employed the SHAP method for a comprehensive interpretability analysis. The SHAP-based feature importance ranking revealed that CRP exerted the greatest average contribution to the model output, followed by LDH, IgA, NEUT, and WBC (Fig 4A). The SHAP summary scatter plot further demonstrated a consistent directional correspondence between distinct feature values and model outputs: higher values of CRP, LDH, IgA, NEUT, and WBC predominantly corresponded to positive SHAP values, suggesting a greater propensity to drive the model prediction towards the occurrence of BP. Conversely, higher values of Zn, Fe, and TBA generally corresponded to negative SHAP values, indicating a tendency to push the prediction towards uncomplicated URTI (Fig 4B).

Local explanations for an identical sample revealed that the directional contributions of features toward BP (Class 1) and uncomplicated URTI (Class 0) exhibited inverse distributions. In the waterfall plots, CRP and WBC showed positive contributions toward BP under the Class 1 display, whereas they showed negative contributions under the Class 0 display (Fig 4C–4D). The force plot results corroborated these findings, illustrating how the combined effects of key features determined the final classification tendency of this specific sample (Fig 4E–4F).

**Fig 5:**
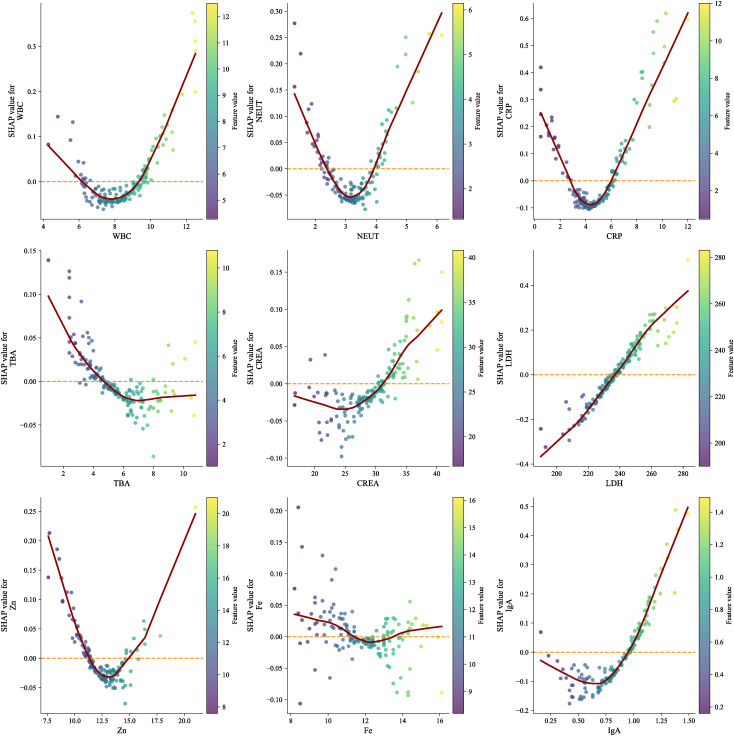
SHAP dependence plots for key features in the SVM model. Each panel depicts the relationship between a feature’s value (x-axis) and its SHAP value (y-axis), characterizing the magnitude and direction of that feature’s contribution to the model output across the observed value range.

Furthermore, SHAP dependence plots indicated nonlinear associations between pivotal variables and the model output. Specifically, the SHAP effects of WBC, NEUT, and CRP showed a “U-shaped” trend as their values fluctuated; LDH generally exhibited an ascending SHAP contribution with increasing measured values, while Zn and IgA also displayed value-interval-dependent effect variations (Fig 5). Overall, the SHAP analysis provided robust evidence of interpretability for the SVM predictions at both the global and individual levels. Multiple laboratory parameters exerted positive or negative predictive momentum across different value intervals, manifesting interval-dependent nonlinear effects in the dependence plots. This ultimately furnished verifiable, biologically plausible interpretability support for the SVM model in discriminating the risk of BP.

## Discussion

Early differentiation of pediatric acute respiratory infections in outpatient and emergency settings often lacks specific clinical phenotypes, subsequently leading to unnecessary empirical antibiotic exposure in children with self-limiting URTI [22,23]. Leveraging a dual-center retrospective cohort, we employed a dimensionality reduction strategy that intersected LASSO regression with the Boruta algorithm to extract nine feature indicators (CRP, LDH, IgA, NEUT, WBC, Zn, CREA, Fe, and TBA) from routine laboratory tests at initial presentation. Based on this feature subset, we constructed and internally evaluated an SVM model tailored to differentiate pediatric BP from uncomplicated URTI.

In current clinical pathways, the early evaluation of pediatric BP primarily relies on clinical signs and radiographic imaging [24]. However, early nonspecific respiratory signs overlap extensively between BP and uncomplicated URTI. Concurrently, chest radiography—the traditional gold standard for definitive diagnosis—is difficult to justify as a routine initial screening tool in emergency and outpatient settings due to inherent ionizing radiation exposure risks [25]. Furthermore, previous auxiliary triage largely relied on isolated inflammatory biomarkers, which offer limited discrimination in reflecting the host’s complex systemic response to infection. Consequently, final clinical decisions are highly sensitive to heterogeneity in attending physicians’ subjective experiences [26,27]. In contrast, by integrating widely accessible, cost-effective routine blood and biochemical laboratory data, we constructed an objective, multidimensional risk-quantification model. This strategy overcomes the suboptimal classification efficacy of traditional single indicators during early differentiation, providing a noninvasive, low-cost auxiliary triage tool to circumvent unnecessary radiographic imaging and empirical antibiotic exposure.

Routine laboratory parameters provide highly valuable objective information for assessing systemic pathology in pediatric patients with acute respiratory infections [28]. The nine feature indicators incorporated into our models encompass three distinct dimensions: immune-inflammatory response, tissue stress, and trace element metabolism. Specifically, alterations in WBC, NEUT, CRP, and IgA directly reflect the host’s acute-phase inflammatory and immune activation levels; abnormalities in LDH, CREA, and TBA indicate local tissue injury and organ metabolic stress secondary to infection; whereas Zn and Fe signify the consumption and redistribution of trace elements during the disease course. A recent large-scale cohort study on pediatric pneumonia confirmed that pathogen-induced systemic inflammation synchronously drives tissue micro-injury and metabolic shifts [29]. Compared to isolated biomarkers, this multidimensional feature panel—spanning “inflammation-injury-metabolism”—more comprehensively captures the pathophysiological cascade in children during the acute infection phase, thereby establishing a robust biological rationale for the model to differentiate BP from uncomplicated URTI [30,31].

In this study, we employed a feature dimensionality reduction strategy derived from the intersection of LASSO regression and the Boruta algorithm. LASSO regression was utilized to mitigate linear multicollinearity among variables, while the Boruta algorithm was applied to extract global nonlinear interactive features; their integration guaranteed the robustness of the final feature subset incorporated into the models. During comparisons in the internal validation set, the SVM model exhibited favorable discrimination and calibration performance. Compared to traditional LR and tree-based models, the SVM algorithm demonstrated superior efficacy in delineating classification boundaries when processing clinical laboratory data characterized by complex nonlinear distributions.

Simultaneously, we quantified the marginal contributions of the nine features to the SVM model’s predictive risk using the SHAP framework. SHAP dependence plots objectively illustrated the nonlinear relationships between routine laboratory parameters and the risk of pneumonia: indicators such as WBC, NEUT, and Zn exhibited a U-shaped distribution in the predictive output, whereas the SHAP values for CRP and LDH showed a continuous positive risk contribution as their measured values increased. This nonlinear quantitative evaluation approach transcends the traditional clinical discrimination paradigm that relies on single, fixed reference intervals (normal vs. abnormal). As delineated in a recent machine learning-based clinical decision support study on pediatric pneumonia by Serin et al., the host’s systemic physiological response to infection rarely exhibits a perfectly linear relationship [32]; the incorporation of the SHAP framework enables the precise estimation of nonlinear risk weights for routine laboratory parameters at specific physiological thresholds, providing a biologically plausible, individualized decision-making basis to break away from the dogmatic use of isolated indicators.

In the DCA, we observed that the model yielded a positive net clinical benefit across a broad range of threshold probabilities. In outpatient and emergency triage settings, it can assist in identifying low-risk pediatric patients with uncomplicated URTI, providing an objective reference to curtail unnecessary antibiotic interventions and ionizing radiation exposure. The limitations of this study are as follows. First, the retrospective design introduces inherent selection bias. In addition, because radiographic confirmation was used as part of the diagnostic criteria for BP, clinicians’ decisions to order chest imaging may have introduced diagnostic selection bias. Therefore, the model may partly reflect clinical decision patterns in retrospective practice rather than purely disease-related biological differences. Future prospective studies with standardized imaging criteria and predefined diagnostic pathways are needed to minimize this bias. Second, although this study included data from two hospitals, data from both centers were merged before random splitting into the training and internal validation sets. Therefore, the reported performance should be interpreted as internal validation based on an internal validation set derived from a merged dual-center cohort, rather than true center-wise external validation. Third, the feature matrix did not include specific etiological sequencing or antimicrobial resistance data. Fourth, the proportion of children younger than 5 years was relatively low in this cohort, which may limit the generalizability of the model to younger pediatric populations. Given the limited sample size of children under 5 years of age, formal age-stratified model performance analysis may yield unstable estimates. Future studies should further validate the model in younger children, particularly those under 5 years of age. Finally, the model’s generalizability requires further validation in independent prospective cohorts, preferably using center-wise external validation strategies. In conclusion, the SVM model, constructed upon nine routine laboratory parameters, effectively evaluates the risk of pediatric BP and holds the potential to be translated into an early auxiliary triage tool in the emergency department.

## Conclusion

This study developed an interpretable machine learning model using nine routine laboratory parameters and internally evaluated its performance in an internal validation set derived from a merged dual-center retrospective cohort, supporting early risk stratification and differentiation between pediatric bronchopneumonia and uncomplicated upper respiratory tract infection. The model provides interpretable quantitative evidence to support initial decision-making in outpatient and emergency settings, offering potential to identify low-risk children and optimize further diagnostic testing and medication strategies.

## Abbreviations

ADA: Adenosine Deaminase
ALB: Albumin
ALP: Alkaline Phosphatase
AUC: Area Under the Curve
BP: Bronchopneumonia
CG: Cholylglycine
CK: Creatine Kinase
CREA: Creatinine
CRP: C-reactive Protein
DCA: Decision Curve Analysis
DT: Decision Tree
EMR: Electronic Medical Record
GGT: Gamma-glutamyl Transferase
GLDH: Glutamate Dehydrogenase
HCY: Homocysteine
IMA: Ischemia-modified Albumin
MONO: Monocyte Count
NEFA: Non-esterified Fatty Acids
NEUT: Neutrophil Count
RBC: Red Blood Cell Count
RBP: Retinol-binding Protein
SA: Sialic Acid
SHAP: Shapley Additive Explanations
TBA: Total Bile Acids
UA: Uric Acid
WBC: White Blood Cell Count
